# Urban Air Quality Shifts in China: Application of Additive Model and Transfer Learning to Major Cities

**DOI:** 10.3390/toxics13050334

**Published:** 2025-04-24

**Authors:** Yuchen Ji, Xiaonan Zhang, Yueqian Cao

**Affiliations:** 1 School of Transportation and Civil Engineering, Nantong University, Nantong 226019, China; jiyc@stmail.ntu.edu.cn (Y.J.); zxn0423666@126.com (X.Z.); 2 State Key Laboratory of Remote Sensing and Digital Earth, Faculty of Geographical Science, Beijing Normal University, Beijing 100875, China

**Keywords:** air quality prediction, generalized additive model, transfer learning, urban pollution

## Abstract

The impact of reduced human activity on air quality in seven major Chinese cities was investigated by utilizing datasets of air pollutants and meteorological conditions from 2016 to 2021. A Generalized Additive Model (GAM) was developed to predict air quality during reduced-activity periods and rigorously validated against ground station measurements, achieving an R^2^ of 0.85–0.93. Predictions were compared to the observed pollutant reductions (e.g., NO_2_ declined by 34% in 2020 vs. 2019), confirming model reliability. Transfer learning further refined the accuracy, reducing RMSE by 32–44% across pollutants when benchmarked against real-world data. Notable NO_2_ declines were observed in Beijing (42%), Changchun (38%), and Wuhan (36%), primarily due to decreased vehicular traffic and industrial activity. Despite occasional anomalies caused by localized events such as fireworks (Beijing, February 2020) and agricultural burning (Changchun, April 2020), our findings highlight the strong influence of human activity reductions on urban air quality. These results offer valuable insights for designing long-term pollution mitigation strategies and urban air quality policies.

## 1. Introduction

Air quality is crucial for human health, e.g., PM_10_ (particulate matter smaller than 10 microns) can penetrate deep into the lungs, causing respiratory issues, blood disorders, and neurodevelopmental problems such as autism, attention deficit disorders, and cognitive delays [1]. Moreover, air pollution negatively impacts cognitive function in the elderly and is linked to higher mortality rates [2]. The economic costs of air pollution are substantial, underscoring the importance of reducing pollution [3], especially in densely populated urban areas. Long-term exposure to PM_10_ and nitrogen dioxide (NO_2_) significantly increases human’s susceptibility to respiratory virus [4] and leads to higher fatality rates among infected individuals [5]. Additionally, emerging evidence suggests that viral particles can be detected in outdoor particulate matter, raising concerns about transmission routes [6]. While further investigations are necessary to fully understand these dynamics, it is increasingly clear that air pollution plays a crucial role in both the transmission and severity of respiratory infections.

During the winter and spring of 2020, most cities in China implemented lockdown measures of various durations and strictness levels to combat the spread of a virus, which proved effective in significantly slowing the transmission of COVID-19 [7,8]. Concurrently, the widespread adoption of reduced mobility prompted researchers to examine the potential benefits of pollution reduction, which is particularly pertinent in the context of global warming, as these shifts in behavior and operations might offer a blueprint for reducing pollution while maintaining the essential functions of cities and nations. The pandemic inadvertently provided a unique opportunity to rethink and reimagine urban planning and environmental policies for a more sustainable future [9,10].

Despite multiple studies that have estimated pollution reductions during lockdowns across different countries [11,12,13,14,15,16], these results often aggregate differences to various baselines without modeling the relationships between air pollution, local weather conditions, time variations, and land-use patterns [17]. On the other hand, the short duration of the lockdowns resulted in a scarcity of representative data, complicating detailed analysis. Moreover, the strict initial lockdown measures in many countries were only implemented for a few weeks, making it difficult to establish effective models for the lockdown period. Addressing these challenges will help accurately measure the reduction in local pollution during the lockdown and understand its spatiotemporal variations, as well as predicting how pollution patterns might change if lockdowns occur in different seasons or are extended in duration.

Generalized Additive Models (GAMs) have been widely adopted in environmental epidemiology and air quality forecasting due to their flexibility in handling non-linear relationships between pollutants and meteorological factors [18,19]. For instance, Pearce et al. [20] demonstrated GAM’s effectiveness in quantifying meteorology’s influence on PM_2.5_ across Australian cities, achieving R^2^ > 0.8 when validated against monitoring stations. Similarly, transfer learning approaches [21] have proven valuable for adapting pre-trained models to new scenarios with limited data, as shown by Bauwens et al. [22] in their analysis of NO_2_ reductions during European lockdowns using satellite-derived datasets. Recent studies highlight the utility of these methods for policy-relevant analysis, which enables disentangling anthropogenic impacts from meteorological variability—a critical need identified by Venter et al. [14] in their global assessment of lockdown effects. Our work extends these applications by integrating transfer learning to address data scarcity during abrupt activity reductions, a methodological advance validated against ground measurements from seven Chinese megacities. The choice of GAM was motivated by its demonstrated success in handling log-normal pollutant distributions [23], accommodating temporal autocorrelation via spline functions [18], and providing interpretable smooth terms for policy design [19].

This study developed a predictive model (LD model) to analyze air quality during periods of reduced human activity in Chinese cities with three specific objectives: (1) establishing a GAM-based pre-LD model using 2016–2019 historical weather and pollution data, (2) employing transfer learning to adapt predictions for lockdown conditions through parameter refinement, and (3) quantifying pollutant variations (NO_2_, CO, PM_10_) across seven cities through comparison of model outputs with ground-station measurements. The framework addresses key challenges in air quality analysis during atypical activity reductions while maintaining model interpretability for policy applications.

## 2. Study Area and Datasets

### 2.1. Study Area

The study area encompasses seven Chinese cities: Beijing, Changchun, Chongqing, Guangzhou, Hangzhou, Wuhan, and Xiamen. Boasting substantial populations, high levels of urbanization and robust economic prowess, these metropolises represent major urban centers across different geographical regions and experience significant daily traffic volumes leading to motor vehicle emissions that impact local air quality. Notably, Changchun is a prominent industrial hub in China where industrial emissions significantly contribute to urban air pollution. The onset of stringent lockdown measures in early 2020 resulted in a marked reduction in traffic flow and industrial emissions across various cities in China—providing an opportunity for our research endeavors. Consequently, these seven cities were strategically selected as representatives for studying the influence of the epidemic-induced lockdown on Chinese urban air quality.

### 2.2. Datasets

Training pre-LD and LD models utilized hourly datasets of air pollutants and meteorology conditions from 1 January 2016 to 31 December 2021 across the aforementioned seven Chinese cities with one site designated per city. The lockdown period is from 23 January to 7 April 2020. Air pollutant data, encompassing NO_2_ concentration (*µ*g/m^3^), CO concentration (*µ*g/m^3^), and PM_10_ concentration (*µ*g/m^3^), as well as meteorological records, including temperature *T* (°C), pressure *P* (hPa), relative humidity *RH* (%), wind direction *WD* (°), and wind speed *WS* (m/s), were both sourced from the China Meteorological Data Center.

## 3. Methods

**Figure 1:**
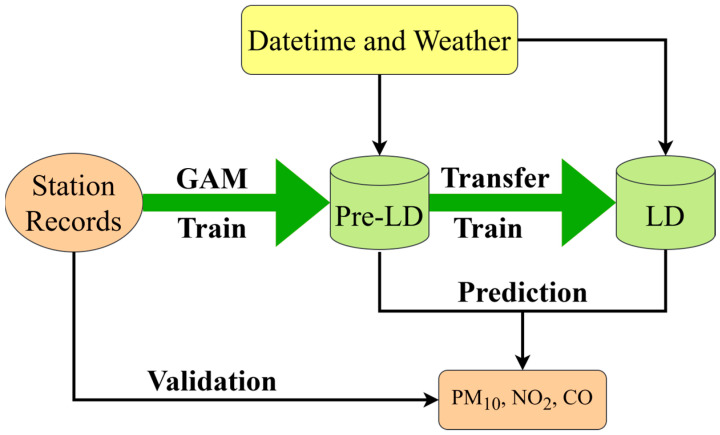
Workflow for air quality analysis during lockdown periods. Station records, including PM_10_, NO_2_, and CO concentrations, along with datetime and weather variables, were input into the pre-LD and LD phases.

To estimate air pollution levels in Chinese urbans unaffected by pandemic-related lockdown measures, the pre-LD and LD models should be with high accuracy and robust predictive power for data forecasting during the lockdown period, while also maintaining a high level of interpretability. A more interpretable model facilitates better comprehension of the impact of traffic patterns on air quality, enabling effective measures to mitigate air pollution through adjustments in relevant traffic flows. Previous studies [18,19,20] have demonstrated the success of GAM in predicting air pollution. Therefore, as illustrated by Figure 1, this work adopted the GAM as the pre-lockdown module. Subsequently, a transfer learning mechanism was employed for training the LD model. Given variations in pollutant concentrations across different regions, certain parameters were adjusted and optimized to enhance prediction accuracy.

### 3.1. Pre-LD Model

Hastie et al. [24] expanded the application of additive models [25] to include the Generalized Additive Model (GAM) as a versatile and flexible statistical tool for identifying non-linear regression effects:

(1) $$g\left(\mu \right)=s_{1}\left(x_{1}\right)+s_{2}\left(x_{2}\right)+s_{3}\left(x_{3}\right)+\cdots +s_{p}\left(x_{p}\right)$$

where $\mu =E(Y|x_{1},x_{2},x_{3},\cdots ,x_{p})$ and $s\left(\cdot \right)$ represents a non-parametric smooth function such as a smooth spline function, kernel function, or local regression smooth function. The distribution of air pollutant concentration closely follows a lognormal distribution [23]. The non-parametric nature of GAM provides significant flexibility to the model and facilitates the elucidation of nonlinear effects arising from derived variables.

In the context of model selection, this study employs the forward selection method, which has been utilized in related fields of environmental science with promising outcomes [26]. The model incorporates two key indicators: the Akaike Information Criterion (AIC) [27] and the Variance Inflation Factor (VIF) [28].

AIC serves as a standard for assessing the adequacy of statistical model fitting, which is defined as follows:

(2) AIC=2k−ln⁡l

where k is the number of model parameters, and l denotes the maximum value of the model likelihood. A small k indicates a parsimonious model, whereas a large l signifies a precise model. AIC emphasizes the significance of data fitting while endeavoring to mitigate overfitting. Consequently, the preferred model for consideration is the one with the lowest AIC value.

VIF is a number that characterizes the degree of complex collinearity between observations of the independent variable as follows:

(3) $$VIF=\frac{1}{1-R_{i}^{2}}$$

where $R_{i}^{2}$ marks the coefficient of determination for the regression analysis of the *i*-th variable with all other explanatory variables. Multicollinearity is a linear or approximate linear relationship between regression variables. The general criteria of VIF are: 0 < VIF ≤ 5, no multicollinearity; 5 < VIF ≤ 10, weak multicollinearity; 10 < VIF ≤ 100, moderate or strong multicollinearity; VIF > 100, severe multicollinearity. The VIF threshold is typically set at 2.5 for processing meteorological data [29]. Thus, this study excluded variables with VIF > 2.5.

For each explanatory variable, a GAM containing only one variable is fitted, and the model with the lowest AIC was chosen. Subsequently, an iterative process was employed to identify the next optimal variable to be added to the existing model. In order to accommodate the weekly variations in pollutants resulting from the pandemic lockdown, the explanatory variable ‘weekday’ was artificially incorporated into cities where it had not been selected.

### 3.2. LD Model

The pre-LD model (GAM) was trained using the pre-LD data. Tied to the limited duration of the lockdown, it is unfeasible to employ the same GAM for training during this period; hence, transfer learning [21] was employed. Given the consistent relationship between weather conditions and air pollution, insights gained from studying how weather impacts air pollution prior to the lockdown can be leveraged during this period. In conducting experiments, it becomes imperative to adjust model variables, particularly with regard to the ‘weekday’ variable that signifies fluctuations in traffic intensity—a pivotal factor influencing air pollution.

## 4. Results and Discussion

### 4.1. The Impact of Lockdown on Air Pollutants and Weather

**Figure 2:**
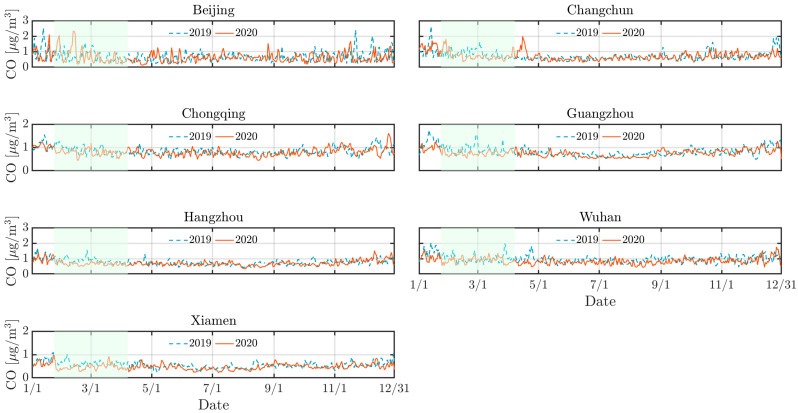
Measurement of CO in different cities across China from 1 January to 30 June in 2019 and 2020. Green zones show the activity-reduction period from 23 January to 7 April 2020.

**Figure 3:**
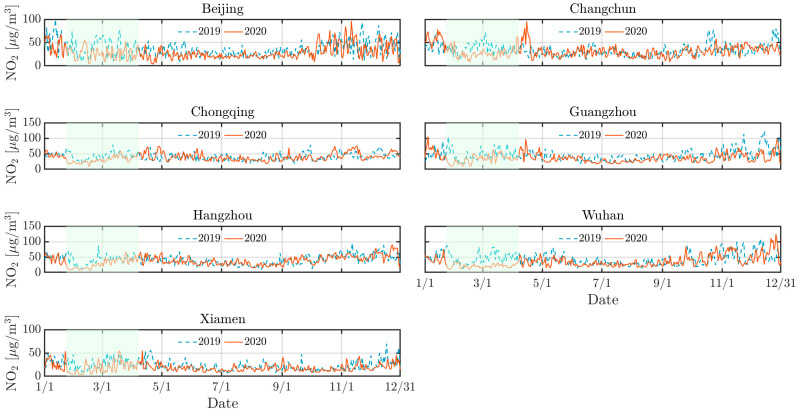
Same as Figure 2 but for NO_2_.

**Figure 4:**
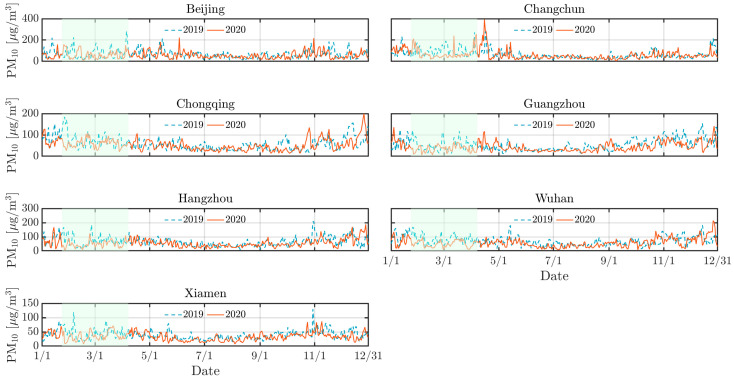
Same as Figure 2 but for PM_10_.

As depicted by Figure 2, Figure 3 and Figure 4, the concentrations of NO_2_, CO, and PM_10_ in the seven cities during the 2020 lockdown were consistently lower than those recorded during the same period in 2019 [30,31]. While the dataset spans 2016–2021 and were used for training and validating the predictive models, only the 2019–2020 period was visualized to highlight year-over-year changes. Quantitatively, NO_2_ levels declined by 42% in Beijing, 38% in Changchun, and 36% in Wuhan, while PM_10_ decreased by 25–35% across most cities. CO concentrations also showed moderate reductions ranging from 10% to 20%. The reduction highlights the significant impact of decreased human activities, such as reduced vehicular traffic and industrial operations, on mitigating air pollution in Chinese urban areas. Particularly, there was a significant reduction in NO_2_ levels in Beijing, Changchun, and Wuhan during the lockdown (Figure 3). NO_2_ primarily originates from automobile exhaust [32] and industrial emissions, and Beijing experienced prolonged and widespread traffic congestion [33]. The substantial decrease in traffic volume during the lockdown resulted in a marked decline of NO_2_ levels. Changchun’s extensive industrial emissions were notably reduced as a result of the lockdown measures, leading to a direct decrease in nitrogen dioxide emissions. In comparison to other cities, Wuhan’s stringent lockdown measures significantly lowered its pollutant concentration.

However, despite the overall decline in pollutant levels, there were notable exceptions. For instance, a marked increase in pollutant concentrations was observed in Beijing in 2020 mid-February, with concentrations temporarily exceeding 250 *µ*g/m^3^. This anomaly can be attributed to the extensive use of fireworks during the Spring Festival, which significantly increased PM_10_ concentrations due to combustion-derived particulate emissions [34]. While fireworks may minimally elevate other pollutants like NO_2_ or SO_2_, their impact is negligible compared to PM_10_ in this context. Furthermore, low wind speeds during this period exacerbated the situation by trapping pollutants close to the ground, further intensifying air quality issues. Similarly, a significant rise in pollutant levels occurred in Changchun in 2020 early April was attributed to the burning of agricultural straw in rural areas surrounding the cities [35], which released substantial quantities of particulate matter and other pollutants into the atmosphere. The reduction in pollution levels during the lockdown provides a compelling argument for the potential long-term benefits of sustained reductions in human activities. Nevertheless, the occasional spikes in pollutant concentrations during specific events highlight the need for targeted air quality management strategies that address both routine and exceptional situations of pollution.

**Figure 5:**
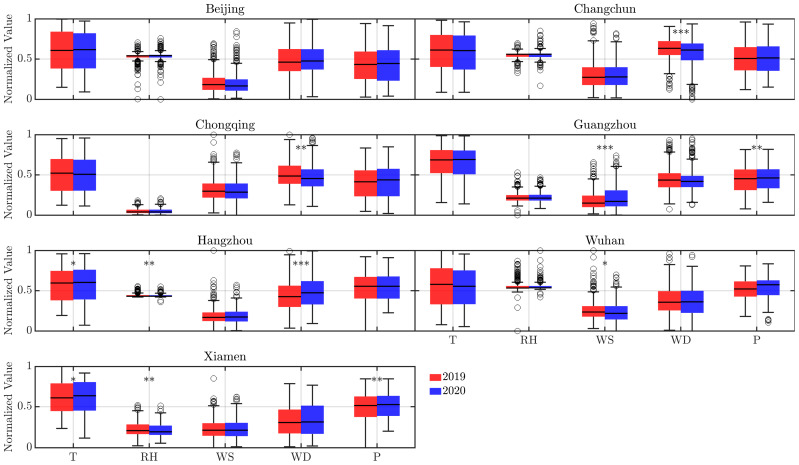
Comparison of normalized meteorological variables between the activity-reduction period in 2020 and the counterpart in 2019 across cities. * denotes statistically significant differences between the two years under paired *t*-test: * for *p*  <  0.05, ** for *p*  <  0.01, *** for *p*  <  0.001.

To further assess the role of meteorological changes in influencing air quality patterns, paired *t*-test was conducted for each variable across all cities (Figure 5), which reveals that relative humidity (*p* < 0.01) and wind direction (*p* < 0.001) exhibited the most consistent and statistically significant differences between 2019 and 2020. These two parameters likely played dominant roles in modulating pollution levels, as consistent humidity and wind direction in 2020 may have limited vertical mixing and horizontal dispersion of pollutants during the lockdown period. Overall, a significant majority of cities experienced cooler temperatures during the lockdown in 2020 compared to the same period in 2019. While reduced human activity may have contributed to a weaker urban heat island effect [36], pollutant concentrations are also strongly influenced by atmospheric stability and boundary layer dynamics, which can vary unpredictably. Meteorological variables (temperature, pressure, dew point, wind speed/direction) were measured at ground-level stations from the China Meteorological Data Center (Section 2.2). Notably, the observed lower temperatures, slower wind speeds, and higher air pressure in 2020 likely suppressed vertical mixing, further inhibiting pollutant dispersion.

### 4.2. Model Predictions

**Figure 6:**
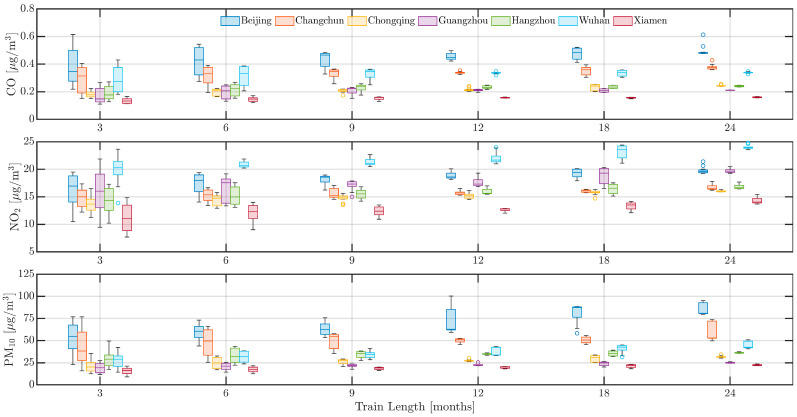
Performance of the pre-LD model depending on training data with different lengths.

The pre-LD model’s predictions utilized data from training periods of 3, 6, 9, 12, 18, and 24 months prior to the lockdown, with subsequent testing against the next month’s data. As shown by Figure 6, the model’s performance improves with shorter training durations, achieving optimal results when trained on 3 months of data. For example, NO_2_ RMSE in Beijing dropped from 19.78 *µ*g/m^3^ (24-month training) to 16.06 *µ*g/m^3^ (3-month training), while PM_10_ RMSE in Changchun decreased from 60.48 *µ*g/m^3^ to 42.77 *µ*g/m^3^. This indicates that a shorter training period allows the model to better capture complex temporal patterns, enhancing predictive accuracy. However, the increased RMSE of PM_10_ in Beijing, despite extended training, can be attributed to the frequent occurrence of sand and haze weather, leading to extreme PM_10_ values prior to the lockdown [37]. The inclusion of these extreme values during the extended training period influenced the model’s accuracy. Table 1 further reveals that the choice of training duration substantially influenced the model performance across different cities and pollutants. One-way ANOVA results demonstrate statistically significant differences in RMSE values for several pollutant–city pairs, underscoring the critical role of temporal sampling in model calibration. Notably, cities such as Chongqing and Xiamen exhibited high sensitivity in CO prediction accuracy across training lengths, suggesting that areas with various emission patterns may benefit from extended training periods. These findings emphasize the need to tailor model training strategies based on local pollutant characteristics and temporal dynamics, which can enhance the robustness and generalizability of predictive frameworks under varying environmental conditions.

Overall, training on 24 months of data generally provided better outcomes, as it offered a richer context for predicting future conditions. This is consistent with the statement that extended training periods promote model performance by incorporating more comprehensive historical data [38].

Due to the scarcity of data during the lockdown period, only three consecutive days of data were used as the test set in the experiment, and the remaining data during the lockdown were used to train the LD model. Table 2 provides a summarized performance of pre-LD and LD models in predicting air pollutant concentrations (NO_2_, CO, and PM_10_) across seven cities. The LD model outperformed the pre-LD model in nearly all cases, particularly for NO_2_ and PM_10_. For example, in Wuhan, the LD model reduced RMSE from 21.87 *µ*g/m^3^ (pre-LD) to 9.03 *µ*g/m^3^ for NO_2_, and from 36.13 *µ*g/m^3^ to 24.20 *µ*g/m^3^ for PM_10_. Among the seven cities, Xiamen’s coastal location, high temperatures, strong wind speeds, and open terrain promote the dispersion of air pollutants. The city also experiences low industrial emissions and maintains smooth traffic year-round. Consequently, Xiamen’s air quality remains generally high on non-lockdown days [39]. As a result, the level of air pollution blocked by the epidemic decreased less than usual, and the predictions of the pre-LD and LD models are more accurate than those of the other six cities. Generally, the RMSE of CO exhibits a significantly lower value against the ones of the other two pollutants and there was an average reduction of 14% for CO, 34% for NO_2_, and 28% for PM_10_ across the seven cities when comparing 2020 lockdown data to 2019 baseline measurements. In contrast to the pre-LD model, the LD one estimated a reduction of 44%, 32%, and 30% in the average RMSE for NO_2_, CO, and PM_10_ predictions in the seven cities, respectively, validating the effectiveness of transfer learning in low-data scenarios.

**Figure 7:**
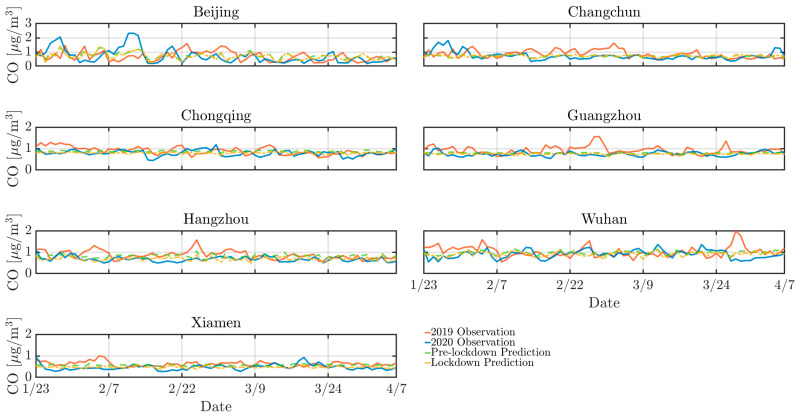
Pre-LD and LD model predictions for CO against 2019 and 2020 observations across cities.

**Figure 8:**
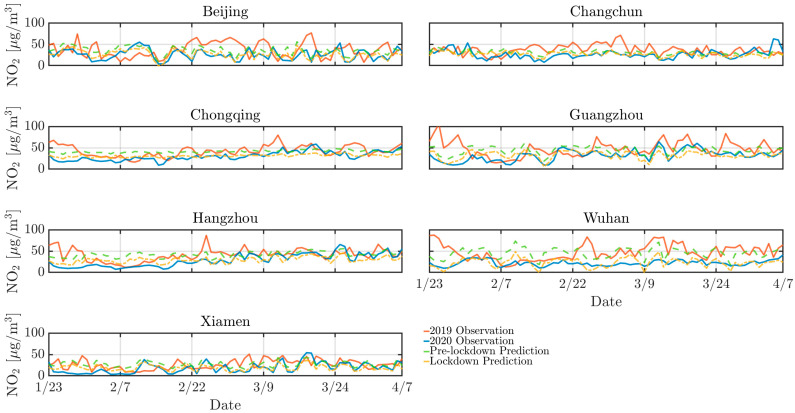
Same as Figure 7 but for NO_2_.

**Figure 9:**
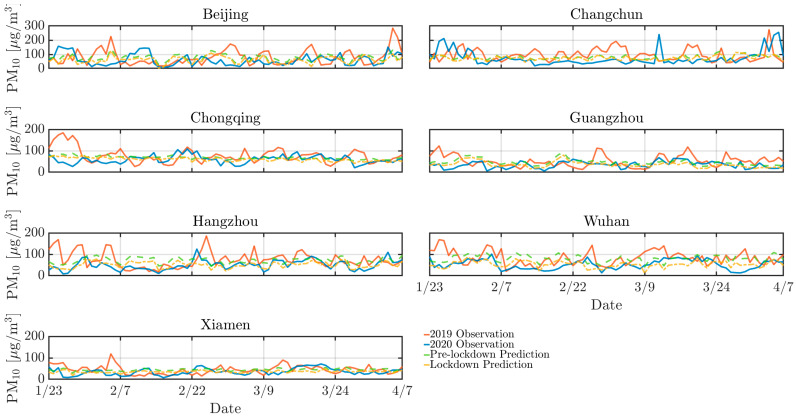
Same as Figure 7 but for PM_10_.

Similarly, Figure 7, Figure 8 and Figure 9 support this behavior, proving that the LD model predicted more accurately than the pre-LD model. The discrepancy between the predicted and the actual values is notably reduced with the LD model, indicating enhanced predictive accuracy. This improved performance can be attributed to the transfer learning that is crucial for enhancing prediction accuracy. However, Figure 7 reveals a significant increase in CO concentrations during the 2020 winter in Beijing and Changchun, leading to considerable deviations between the LD model’s predictions and actual observed values. This discrepancy is likely because of higher CO emissions resulting from increased heating activities in northern cities during the winter [36,40]. Moreover, pollution levels were generally higher in 2019 than 2020, which can be explained by the higher emissions of air pollutants resulting from much larger traffic volumes prior to the lockdown in the seven cities. In contrast, the lockdown period, with its restrictions on movement and reduced industrial activity, contributed to a significant improvement in overall air quality.

Table 3 presents the agreement between the LD model-predicted NO_2_ reduction and the measurements from satellite images. The model’s predictions fall within a reasonable range, with those for Beijing (−35% vs. −25% and −33%, respectively) and Wuhan (−47% vs. −43% and −57%) aligning closely with observations from both satellites. Moderate deviations were observed in Chongqing and Guangzhou but the estimates remained within the uncertainty ranges of satellite products. This validation against independent data sources affirms the robustness of our modeling framework and highlights its applicability for real-time or retrospective air quality assessments when ground-based data are limited.

## 5. Conclusions

This study evaluated the impact of reduced human activity on urban air quality across seven major Chinese cities during the COVID-19 lockdown period via a modeling framework that combined GAM and transfer learning. The findings show that, compared to the same period in 2019, concentrations of NO_2_, CO, and PM_10_ declined by an average of 34%, 14%, and 28%, respectively, during the 2020 lockdown. The most substantial reductions in NO_2_ were observed in Beijing (−42%), Changchun (−38%), and Wuhan (−36%), largely due to decreased vehicular traffic and industrial activity.

Model performance improved with decreased training duration, with RMSE decreasing by up to 50% when models were trained on 3 months of historical data. Transfer learning significantly enhanced the prediction accuracy under limited-data conditions, with RMSE reductions of 44% for NO_2_, 32% for CO, and 30% for PM_10_ across all the cities. These results were validated against satellite observations, showing close agreement and confirming the robustness of the modeling framework.

This study demonstrates the value of combining interpretable statistical models with transfer learning to predict urban air quality under abrupt activity changes. The insights are directly relevant to environmental policy, showing that substantial improvements in air quality can be achieved through targeted reductions in anthropogenic emissions. The modeling approach also offers a transferable methodology for assessing future emission-control strategies under both planned and unexpected disruptions.

## Figures and Tables

**Table 1:** Mean training RMSE of the three pollutants across training lengths for all cities. * marks statistically significant differences among durations based on one-way ANOVA: * for *p*  <  0.05, ** for *p*  <  0.01, *** for *p*  <  0.001.

Duration [Months]	Beijing			Changchun			Chongqing			Guangzhou			Hangzhou			Wuhan			Xiamen		
	CO *	NO_2_ ***	PM_10_ ***	CO **	NO_2_ ***	PM_10_ *	CO ***	NO_2_ ***	PM_10_ ***	CO *	NO_2_ ***	PM_10_ ***	CO **	NO_2_ ***	PM_10_	CO	NO_2_ ***	PM_10_ ***	CO ***	NO_2_ ***	PM_10_ ***
3	0.39	16.06	52.94	0.29	14.81	42.77	0.18	13.66	21.46	0.17	15.82	19.13	0.19	14.34	29.75	0.28	19.78	28.11	0.13	11.19	15.62
6	0.42	17.29	59.02	0.32	15.28	47.87	0.20	14.39	24.75	0.20	16.52	20.69	0.21	15.18	32.94	0.31	20.88	31.66	0.15	11.97	17.45
9	0.44	18.16	62.93	0.33	15.61	50.03	0.21	14.78	26.20	0.21	17.07	21.70	0.23	15.49	34.25	0.33	21.30	34.04	0.15	12.37	18.59
12	0.46	18.86	72.76	0.34	15.74	50.14	0.21	15.16	27.35	0.21	17.59	22.53	0.23	15.92	34.80	0.34	22.03	36.77	0.16	12.64	19.47
18	0.48	19.27	81.34	0.35	16.05	50.74	0.23	15.80	29.76	0.21	18.76	23.87	0.23	16.44	35.44	0.33	23.22	41.02	0.16	13.32	21.24
24	0.50	19.78	85.90	0.38	16.72	60.48	0.24	16.03	31.72	0.21	19.72	24.95	0.24	16.83	36.26	0.34	24.00	45.20	0.16	14.29	22.31

**Table 2:** Performances of the pre-LD and LD model in 10-fold cross-validation.

Model	Measure	Beijing			Changchun			Chongqing			Guangzhou			Hangzhou			Wuhan			Xiamen		
		CO	NO_2_	PM_10_	CO	NO_2_	PM_10_	CO	NO_2_	PM_10_	CO	NO_2_	PM_10_	CO	NO_2_	PM_10_	CO	NO_2_	PM_10_	CO	NO_2_	PM_10_
pre-LD	RMSE [*µ*g/m^3^]	0.44	18.24	69.15	0.34	15.70	50.34	0.21	14.97	26.87	0.20	17.58	22.14	0.22	15.70	33.91	0.32	21.87	36.13	0.15	12.63	19.11
	n-RMSE	0.046	0.073	0.011	0.034	0.063	0.008	0.022	0.060	0.004	0.020	0.071	0.004	0.023	0.063	0.005	0.033	0.088	0.006	0.015	0.051	0.003
LD	RMSE [*µ*g/m^3^]	0.41	9.07	40.45	0.22	7.40	42.29	0.14	8.16	20.02	0.10	10.23	16.28	0.13	11.19	18.96	0.19	9.03	24.20	0.12	8.66	14.58

**Table 3:** Estimated and satellite-observed NO_2_ reduction in Beijing, Chongqing, Guangzhou, and Wuhan.

Estimators	Beijing [%]	Chongqing [%]	Guangzhou [%]	Wuhan [%]
LD	−35	−18	−17	−47
TROPOMI [22]	−25 (±10)	−43 (±14)	−30 (±14)	−43 (±14)
OMI [22]	−33 (±10)	−11 (±32)	−56 (±8)	−57 (±14)

## Data Availability

All data generated or analysed during this study are included in this published article.
